# Immune Cells in the Tumor Microenvironment: Mechanisms of Immune Resistance and Therapeutic Advances

**DOI:** 10.2174/0113895575434666260127052401

**Published:** 2026-04-16

**Authors:** Rongfan Tang, Chaoqi Liu, Yafei Zhang

**Affiliations:** 1 The First Affiliated Hospital and College of Clinical Medicine of Henan University of Science and Technology, Luoyang, 471003, China;; 2 Hubei Key Laboratory of Tumor Microenvironment and Immunotherapy, China Three Gorges University, Yichang, Hubei, 443002, China;; 3 Medical College of China Three Gorges University, Yichang, Hubei, 443002, China

**Keywords:** Tumor microenvironment (TME), immune resistance, tumor-associated macrophages (TAMs), Regulatory T cells (Tregs), myeloid-derived suppressor cells (MDSCs), extracellular matrix (ECM)

## Abstract

The Tumor Microenvironment (TME) is a complex and dynamic network composed of immune cells, stromal cells, and the Extracellular Matrix (ECM), which collectively influence tumor progression, metastasis, and resistance to various therapies, ultimately shaping clinical outcomes. Crosstalk between tumor cells and these microenvironmental components fosters immune evasion, metabolic reprogramming, and resistance to immunotherapy. Accumulating evidence indicates that Tumor-Associated Macrophages (TAMs) often polarize toward immunosuppressive M2 phenotypes, secreting cytokines like IL-10 and TGF-β that dampen immune responses. Regulatory T cells (Tregs) inhibit effector T-cell activation, while Myeloid-Derived Suppressor Cells (MDSCs) impair T-cell and NK-cell functions *via* the release of suppressive mediators such as arginase and reactive oxygen species. Additionally, Natural Killer (NK) cells frequently lose cytotoxic potential in the TME. These immunosuppressive mechanisms collectively contribute to resistance against immune checkpoint inhibitors and other immunotherapies. To counteract this, emerging strategies aim to reprogram the TME and enhance anti-tumor immunity. These include the use of cytokine modulators to reverse immunosuppression, adoptive cell therapies such as CAR-T and CAR-NK cells to target tumors directly, and ECM-targeted therapies to improve immune infiltration. This review integrates the current understanding of how the TME mediates immune resistance and explores innovative therapeutic interventions aimed at overcoming these barriers to restore effective anti-tumor immune responses.

## 1 INTRODUCTION

TME is a complex cellular environment, which consists of many types of cells and the extracellular components surrounding tumor cells. Tumor cells, surrounding blood arteries, cytokines, immune cells, stromal cells, and extracellular matrix are some of the common features of the TME, despite the fact that its specific components vary and are dependent on the kind of tumor [1]. The cellular as well as extracellular components of the TME interact with tumor cells, promote tumor proliferation as well as invasion, and decrease drug permeability [2, 3]. Immune cells are the key components of the TME [4]. Immune cell interactions within the TME mediate antitumor responses (Fig. **1**).

Tumor drug resistance is a major challenge in cancer treatment, which can cause treatment failure as well as disease progression. The TME induces chemoresistance through acquired or de novo mechanisms. In acquired Multi-Drug Resistance (MDR), the expression of ATP-Binding Cassettes (ABCs), oncogene activation, and tumor-suppressor gene deregulation occur *via* cellular crosstalk and cell-to-TME interactions. Cancer cells that have been exposed before have phenotypic alterations that make them resistant to further therapies [5]. However, it has been demonstrated that stromal tissue in the TME shields a subset of cancer cells and confers chemoresistance through stemness following therapeutic exposure [6]. In this review, we reviewed the connection of the TME with tumor development or tumor drug resistance, with a particular emphasis on TAMs, Tregs, MDSCs, Natural Killer cells (NK cells), as well as ECM. Specifically, we focus on recent progress in reversing the immune resistance of tumor cells and delve into novel strategies for overcoming drug resistance. The Tumor Immune Microenvironment (TME) plays a pivotal role not only in tumor progression but also in the development of resistance to immunotherapy, and has emerged as a key target in anti-cancer strategies [7].

## 2 METHODS

### 2.1 Literature Search Strategy

A comprehensive literature search was conducted across three major databases: PubMed, Web of Science, and Google Scholar, up to 2025. The search strategy combined Medical Subject Headings (MeSH) terms and free-text keywords, focusing on the tumor microenvironment, immune resistance, and specific immune cell subsets. The search framework was based on three concept groups combined using Boolean operators: (1) tumor microenvironment-related terms (*e.g.*, "tumor microenvironment", "TME", "tumor immune microenvironment"); (2) immune resistance and therapy-related terms (*e.g.*, "immune resistance", "immunotherapy resistance", "drug resistance", "immune evasion", "checkpoint blockade"); and (3) specific immune cell populations (*e.g.*, "tumor-associated macrophages", "regulatory T cells", "myeloid-derived suppressor cells", "natural killer cells", "tumor-associated neutrophils", "extracellular matrix"). Additionally, targeted searches were performed for specific resistance mechanisms, including antigen presentation defects (*e.g.*, "MHC class I", "TAP-1", "β2-microglobulin"), adoptive cell therapy resistance (*e.g.*, "CAR-T", "CAR-NK", "TIL therapy"), metabolic reprogramming (*e.g.*, "metabolic reprogramming", "glycolysis"), and epigenetic regulation (*e.g.*, "m6A methylation", "METTL3", "YTHDF2"). Furthermore, manual citation tracking was performed on highly influential articles and frequently cited reviews to supplement electronic searches and capture important studies not covered by database searches.

### 2.2 Eligibility Criteria

Inclusion criteria: Studies were included if they: (1) investigated immune cell-mediated mechanisms of tumor progression, immune evasion, therapeutic resistance, or immune suppression in the tumor microenvironment, (2) provided mechanistic evidence, such as molecular signaling pathways involved in immune resistance, metabolic reprogramming of tumor or immune cells, cell-cell or cell-matrix interactions, or epigenetic/transcriptional regulation of immune suppression, (3) evaluated therapeutic strategies targeting immune components of the TME, including immune checkpoint inhibition, adoptive cell therapies (CAR-T, CAR-NK, TIL), immunomodulatory agents, or combination therapies, (4) were peer-reviewed original research articles, reviews, systematic reviews, or meta-analyses, and (5) were published in English. Exclusion criteria: Studies were excluded if they: (1) were conference abstracts, editorials, commentaries, opinion pieces, or other publications without original data, (2) were purely descriptive studies lacking mechanistic investigation, (3) had incomplete methodological details or insufficient information to assess study validity, (4) were duplicate publications or preliminary versions superseded by complete studies, and (5) focused exclusively on non-immune components without addressing immune cell regulation.

### 2.3 Data Extraction and Synthesis

Titles and abstracts of all retrieved articles were initially screened to identify potentially relevant studies, followed by independent assessment of full-text articles for eligibility. For each included study, the following information was systematically extracted: (1) study characteristics including first author, publication year, tumor type, experimental model (*in vitro*, animal models, patient samples, clinical studies), and sample size, (2) immune cell populations investigated, including primary cell types (TAMs, Tregs, MDSCs, NK cells, TANs, *etc*.), phenotypic markers and functional properties, and distribution/abundance within the microenvironment, (3) immune resistance mechanisms, including specific signaling pathways (*e.g.*, STAT3, NF-κB, PI3K/AKT, Wnt/β-catenin), secreted suppressive mediators (cytokines, chemokines, metabolites), cell-cell interactions, metabolic alterations (glycolysis, lipid metabolism, amino acid metabolism), and epigenetic modifications (DNA methylation, histone modifications, RNA modifications), (4) relationship to therapeutic resistance, including therapy types (chemotherapy, immunotherapy, targeted therapy, radiotherapy), mechanistic evidence linking immune cells to resistance, and supporting evidence from preclinical models or clinical cases, and (5) therapeutic targeting strategies, including intervention targets, combination regimens, efficacy data, and clinical trial information where available.

Evidence from included studies was synthesized qualitatively, organized thematically by immune cell type (TAMs, Tregs, MDSCs, NK cells, TANs) and structural components (ECM). Findings were further categorized into tumor-intrinsic immune evasion mechanisms (*e.g.*, MHC-I downregulation, TAP loss, β2-microglobulin mutations) and extrinsic immunosuppressive mechanisms mediated by recruited immune cells. Cross-cell-type common patterns were identified, including cytokine signaling, metabolic reprogramming, and checkpoint molecule expression, to construct an integrated mechanistic framework of immune resistance in the TME. Evidence on emerging therapeutic strategies (*e.g.*, CAR-T/CAR-NK therapies, metabolic modulators, epigenetic regulators, and ECM targeting) was also synthesized thematically. Throughout the synthesis process, attention was paid to identifying convergent mechanisms across cell types to reveal shared resistance pathways and potential therapeutic targets.

## 3 RESULTS

### 3.1 Literature Search and Study Characteristics

A comprehensive literature search identified relevant evidence on immune cell-mediated resistance mechanisms in the tumor microenvironment. The included literature comprised original research articles and reviews published predominantly in recent years. Studies encompassed diverse tumor types, with frequently investigated malignancies including non-small cell lung cancer, melanoma, breast cancer, colorectal cancer, pancreatic cancer, and gastric cancer. The evidence primarily examined tumor-associated macrophages, regulatory T cells, myeloid-derived suppressor cells, natural killer cells, and tumor-associated neutrophils, collectively offering mechanistic insight into how distinct immune subsets contribute to therapeutic resistance and how these pathways may be exploited for intervention.

### 3.2 Hierarchical Organization of Immune Resistance Mechanisms

The synthesized evidence revealed that immune resistance in the TME operates through a hierarchical organization. At the foundational level, tumor-intrinsic molecular defects—including MHC class I loss, TAP-1/TAP-2 downregulation, and β2-microglobulin mutations—fundamentally impair antigen presentation, rendering tumor cells invisible to cytotoxic T lymphocytes. These molecular defects create a permissive microenvironment with reduced inflammatory signaling, facilitating the recruitment and activation of immunosuppressive cell populations. At the second level, recruited immunosuppressive cells amplify resistance through multiple interconnected mechanisms. Tumor-associated macrophages, particularly M2-polarized subsets, promote resistance by secreting IL-10 and TGF-β, activating STAT3 and JAK1/STAT3 signaling pathways, inducing METTL3-mediated m6A RNA methylation, and transferring GATA3 *via* extracellular vesicles to upregulate checkpoint molecules. Regulatory T cells sustain immunosuppression through CCR4-mediated recruitment, YTHDF2/m6A/NF-κB axis activation, and IL-10-induced PD-L1 expression on myeloid cells. Myeloid-derived suppressor cells directly inhibit T-cell and NK-cell function *via* ROS, nitric oxide, and arginase secretion, while promoting Treg expansion and exacerbating tumor hypoxia. Natural killer cells exhibit functional exhaustion and metabolic fitness loss due to nutrient competition with tumor cells. At the third level, structural components reinforce immunosuppression. The extracellular matrix serves as both a physical barrier and an active signaling platform that reprograms infiltrating macrophages. Tumor-associated neutrophils mediate resistance through neutrophil extracellular traps, which activate TGF-β signaling and suppress T-cell proliferation.

### 3.3 Convergent Mechanisms and Therapeutic Implications

Synthesizing evidence across immune subsets revealed several convergent mechanisms underpinning resistance. Analysis across immune cell types identified several convergent mechanisms: IL-10 and TGF-β as master immunosuppressive cytokines; multi-layered PD-L1 expression on both tumor and myeloid cells; shared metabolic reprogramming toward glycolysis and amino acid depletion; and m6A RNA methylation as a critical regulatory mechanism. These convergent pathways create redundant suppressive circuits that explain the limited efficacy of single-agent therapies. The reviewed literature validated several promising therapeutic approaches. For TAMs, strategies include CSF1R inhibition, CCL2/CCR2 blockade, CAR-T-mediated depletion of FRβ+ TAMs, and M1 repolarization. For Tregs, approaches include CCR4 inhibition, FOXP3-targeting antisense oligonucleotides, and GITR agonists. For MDSCs, strategies include differentiation induction with all-trans retinoic acid, recruitment blockade *via* chemokine receptor inhibitors, and metabolic targeting. For NK cells, enhancement strategies include cytokine modulation, CAR-NK engineering, and STING agonist-loaded nanoparticles. Emerging directions include m6A methylation modulators, metabolic reprogramming agents, extracellular vesicle targeting, and combination approaches addressing multiple resistance layers simultaneously.

## 4 OVERVIEW OF TUMOR IMMUNE MICROENVIRONMENT AND RESISTANCE TO IMMUNOTHERAPY

The mechanisms of immune resistance in the TME can be broadly categorized into two complementary and interconnected categories: tumor-intrinsic molecular mechanisms and cell-mediated immunosuppressive mechanisms. While immunosuppressive cells such as TAMs, Tregs, and MDSCs play crucial roles in creating an immunosuppressive microenvironment, tumor cells themselves possess intrinsic molecular defects that fundamentally disrupt antigen presentation and recognition at the most basic level. These tumor-intrinsic molecular mechanisms, including defects in MHC-I antigen presentation, loss of TAP-1/TAP-2 expression, and β2-microglobulin mutations, often represent the initial layer of immune resistance, occurring at the molecular level within tumor cells and frequently preceding cell-mediated immune suppression.

The tumor immune microenvironment is composed of various cell types and the extracellular matrix and is closely associated with tumor development. Tumor development is a complex process involving multiple genes, multiple steps, and multiple stages, in which the TME and the immune microenvironment play a critical role [4]. The tumor immune microenvironment is modulated by the density, composition, function, and organization of immune cells infiltrating the tumor. These immune cells activate immunosuppressive pathways and produce immune checkpoint receptors, cytokines, and metabolites, which can affect the function of effector T cells and myeloid cells.

The TME is closely related to tumor incidence and development. The imbalance between pro-tumor and anti-tumor factors changes the disease pattern, affects local lesions, and leads to drug resistance [6]. Some infiltrating immune cells stimulate tumor cell proliferation and promote tumor cell metastasis and angiogenesis. The immunosuppressive environment is considered the main factor undermining the efficacy of immune checkpoint inhibitors. Specifically, MDSCs, tumor-associated macrophages, Tregs, and cytokines can inhibit immune-mediated anti-tumor effects [8].

CAR-T cell therapy faces significant challenges due to tumor-intrinsic and extrinsic resistance mechanisms. A major hurdle is antigen escape, in which tumors downregulate or lose target antigens (*e.g.*, in B-cell malignancies and solid tumors), driving relapse [9]. Additionally, tumor immune evasion may arise from impaired antigen-processing mechanisms, such as the downregulation of Transporter Associated with Antigen Processing (TAP-1 and TAP-2), which limits MHC-I presentation and cytotoxic T-cell recognition, as demonstrated in oral squamous carcinoma [10]. In pancreatic cancer, resistance involves both GPI-anchor biosynthesis (antigen-dependent) and death receptor/transcriptional dysregulation (antigen-independent), with TFAP4-NF-κB-PD-L1 axis inhibition proposed as a solution [11]. For CAR-NK cells, advantages include lower toxicity (no CRS/neurotoxicity) and allogeneic potential, but limited persistence remains a barrier [12].

Tumor-Infiltrating Lymphocyte (TIL) Therapy involves isolating, expanding, and reinfusing a patient's own tumor-infiltrating lymphocytes, demonstrating efficacy even in immune checkpoint inhibitor (ICI)-resistant cancers such as Non-Small Cell Lung Cancer (NSCLC) [13]. Yet, challenges remain in overcoming T-cell exhaustion and immunosuppressive elements within the TME, like Cancer-Associated Fibroblasts (CAFs) and TAMs.

### 4.1 Tumor-intrinsic Molecular Mechanisms of Immune Evasion

While the immunosuppressive functions of TAMs, Tregs, and MDSCs represent critical components of immune resistance, tumor cells themselves possess intrinsic molecular mechanisms that contribute fundamentally to immune evasion. These molecular-level mechanisms often represent the initial layer of immune resistance, occurring at the most fundamental level of antigen presentation and recognition. Understanding these mechanisms is essential for a comprehensive understanding of immune resistance, as they frequently precede and enable subsequent cell-mediated immune suppression [14].

#### 4.1.1 Defects in the MHC-I Antigen Presentation Pathway

The major histocompatibility complex class I (MHC-I) antigen presentation pathway serves as the cornerstone of adaptive immune recognition, enabling Cytotoxic T Lymphocytes (CTLs) to identify and eliminate cells expressing foreign or mutated antigens. MHC-I molecules are heterodimers consisting of a polymorphic heavy chain and the invariant β2-microglobulin (β2m) light chain. This complex is essential for presenting intracellular peptides (typically 8-10 amino acids in length) on the cell surface, where they are recognized by CD8+ T cells *via* their T-cell receptors [15].

Tumor cells frequently develop defects in the MHC-I antigen presentation pathway as a primary mechanism of immune evasion through multiple mechanisms: Genetic alterations such as somatic mutations or deletions in MHC-I heavy chain genes (HLA-A, HLA-B, HLA-C) can result in complete loss of MHC-I expression, often associated with poor prognosis and immunotherapy resistance [16]. Epigenetic silencing through DNA methylation or histone modifications allows reversible downregulation of MHC-I [17]. Transcriptional repression by oncogenic signaling pathways, such as Wnt/β-catenin activation, can downregulate MHC-I expression in melanoma and other cancers [18]. These defects render tumor cells "invisible" to CTLs, creating a fundamental barrier to immunotherapy [19].

#### 4.1.2 Loss of TAP-1/TAP-2 Expression and Antigen Processing Defects

The Transporter Associated with Antigen Processing (TAP) complex represents a critical bottleneck in MHC-I antigen presentation. The TAP complex consists of TAP-1 and TAP-2 subunits, which form a heterodimeric transporter in the Endoplasmic Reticulum (ER) membrane, responsible for ATP-dependent transport of proteasome-generated peptides from the cytosol into the ER lumen for MHC-I loading [20]. The antigen presentation process proceeds through multiple coordinated steps: intracellular proteins undergo proteasomal degradation, and the resulting peptides are transported *via* TAP into the ER, where they are trimmed by ER aminopeptidases. Subsequently, MHC-I molecules are loaded with peptides through a process facilitated by chaperones, including calreticulin, ERp57, and tapasin, before being transported to the cell surface *via* the Golgi apparatus [21].

Tumor cells frequently downregulate or lose TAP-1/TAP-2 expression through genetic mutations, resulting in non-functional TAP complexes [22], epigenetic silencing, which provides reversible downregulation mechanisms, and transcriptional repression by oncogenic pathways. Without functional TAP complexes, peptides cannot reach the ER, resulting in either unstable empty MHC-I molecules that fail to reach the cell surface or MHC-I molecules with suboptimal peptides that cannot activate T cells. In oral squamous cell carcinoma and other malignancies, TAP downregulation directly correlates with reduced MHC-I surface expression and impaired CTL recognition [10, 17, 23].

#### 4.1.3 β2-microglobulin Mutations and MHC-I Complex Assembly

β2-microglobulin (β2m) is an essential component of the MHC-I complex, required for stable expression and proper folding of MHC-I heavy chains. The β2m subunit stabilizes the MHC-I heavy chain and is required for peptide binding and cell-surface expression. Without β2m, MHC-I heavy chains cannot form stable complexes and are rapidly degraded in the ER [24].

Mutations in the β2m gene represent another critical immune evasion mechanism. These mutations include point mutations that disrupt β2m function or stability, frameshift mutations resulting from insertions or deletions that produce truncated or non-functional β2m proteins, and complete deletions leading to the absence of β2m expression. The loss of functional β2m prevents MHC-I assembly, resulting in complete failure of antigen presentation. This is particularly significant because β2m mutations are often selected during immunotherapy, providing a survival advantage under CTL pressure. Studies show β2m mutations emerge as resistance mechanisms to immune checkpoint inhibitors and adoptive T-cell therapy [25], with patients showing initial responses followed by resistance development through selection of β2m-deficient clones.

#### 4.1.4 Linkage between Molecular Defects and Immune Resistance: Establishing the Mechanistic Cascade

The molecular defects described above create a cascade of events leading to complete immune resistance. These defects represent the initial and most fundamental layer of immune evasion, often preceding immunosuppressive cell recruitment. For example, TAP downregulation makes tumor cells "invisible" to CTLs even before TAMs, Tregs, or MDSCs are recruited. The failure of antigen presentation creates a permissive microenvironment with reduced pro-inflammatory cytokines (IFN-γ, TNF-α) and chemokines, decreased immune pressure, and diminished danger signals, facilitating the establishment of immunosuppressive functions by recruited immune cells.

The transition from antigen escape to immune-cell-mediated suppression occurs as follows: impaired antigen transport through TAP downregulation or MHC-I loss disrupts the tumor's ability to present antigens to cytotoxic T cells. Even if T cells are activated through vaccination, immune checkpoint blockade, or adoptive transfer, they cannot recognize and eliminate tumor cells. This creates a scenario in which activated but ineffective T cells become exhausted or anergic [26], immune surveillance decreases, and immunosuppressive cells further suppress remaining responses, thereby compounding immune evasion [27]. This creates a "double hit": first, molecular defects prevent antigen recognition; second, immunosuppressive cells suppress any remaining immune activity. This sequential progression—from molecular defects through antigen escape and T-cell dysfunction to establishment of an immunosuppressive microenvironment—represents a hierarchical cascade where each layer of immune evasion reinforces the next. These mechanistic insights suggest that combination therapies targeting both molecular defects and immunosuppressive cells may offer superior therapeutic efficacy compared to single-agent approaches [28].

Having established the foundational role of tumor-intrinsic molecular defects in immune evasion, the following examines the key immunosuppressive cell populations in the TME and their contributions to immune resistance. Importantly, these cell-mediated mechanisms often operate downstream of the molecular defects described above, creating a multilayered system of immune evasion that presents significant therapeutic challenges.

Taken together, current studies indicate that tumor immune resistance arises from the hierarchical interplay between tumor-intrinsic molecular defects and extrinsic immunosuppressive mechanisms within the TME. At the foundational level, molecular defects including MHC-I loss, TAP downregulation, and β2-microglobulin mutations render tumor cells invisible to cytotoxic T lymphocytes, establishing the first layer of immune evasion that operates independently of the cellular microenvironment. This initial escape from immune recognition creates a permissive niche characterized by reduced inflammatory signaling and diminished recruitment of effector immune cells. Subsequently, as immune pressure decreases, immunosuppressive cell populations—including TAMs, Tregs, MDSCs, and dysfunctional NK cells—are preferentially recruited and activated, establishing a second layer of active immune suppression that reinforces and amplifies the effects of tumor-intrinsic defects. This hierarchical organization helps explain why single-agent immunotherapies, which typically target only one layer of immune resistance, often fail to produce durable responses. Moreover, it provides a mechanistic rationale for combination strategies that simultaneously restore antigen presentation (*e.g.*, through epigenetic modulation or interferon signaling), deplete or reprogram immunosuppressive cells, and enhance effector T-cell function through checkpoint blockade or adoptive cell therapy. Understanding this cascade is essential for designing next-generation immunotherapeutic approaches that address the multi-layered nature of immune resistance.

## 5 TUMOR-ASSOCIATED MACROPHAGES AND TUMOR IMMUNE RESISTANCE

### 5.1 Heterogeneity and Plasticity of Tumor-associated Macrophages

TAMs are the main cells in the TME. In many solid tumors, TAMs account for the largest portion of infiltrating immune cells and link inflammation to tumorigenesis [29]. The heterogeneity and plasticity of macrophages play an important part in tumor development as well as drug resistance. TAMs are a unique subset of peripheral blood monocyte-derived macrophages that move to tumor tissues. They are a crucial part of the TME, creating an immunosuppressive microenvironment that aids in the growth and spread of tumors [30]. Usually, TAMs can differentiate into one of two activation states: M1 macrophages, which repress tumors, or M2 macrophages, which promote tumors [31]. M1 macrophages destroy tumor cells, promote inflammation, and stop tumor growth. Conversely, M2 promotes tumor development, invasion, metastasis, and angiogenesis by suppressing inflammatory processes and modifying immune cells [32]. M1 macrophages use antibody-dependent cell-mediated cytotoxicity to destroy cancer cells. They generate inflammatory cytokines, TNF-α, and chemicals that destroy tumor cells [33]. In contrast, activated M2 macrophages promote angiogenesis and suppress immune cells' anticancer activity, thereby accelerating tumor growth, invasion, and spread [34].

### 5.2 Mechanism of Drug Resistance Mediated by TAMs

Recent findings indicate that immunosuppressive cells such as TAMs, MDSCs, and Tregs secrete factors like TGF-β and IL-10 to reshape the TME, contributing significantly to therapy resistance across multiple cancer types [7]. TAMs mediate resistance to chemotherapy and radiation therapy through activating the signal transducer as well as activator of transcription 3 (STAT3) signaling pathway [35]. Wang *et al.* [36] found that TAMs enhance cancer stemness and the resistance of Hepatocellular Carcinoma (HCC) cells to the first-line drug sorafenib by activating the CXCR2/ERK signaling pathway through paracrine release of the chemokines C-X-C motif chemokine ligand 1 (CXCL1) and CXCL2. Wu *et al.* [37] reported that M2-polarized Tumor-Associated Macrophages (M2-TAMs) contribute to immunotherapy resistance in NSCLC by enhancing METTL3-mediated m6A RNA methylation. M2-TAMs secrete IL-10 as well as TGF-β1, which induce METTL3 expression in tumor cells, enhance m6A modification, and impair CD8⁺ T cell-mediated cytotoxicity. Targeting METTL3 effectively restored antitumor immune responses. Wu *et al.* [38] demonstrated that upregulation of TGF-β1 and valyl-tRNA synthetase 1 (VARS1) in M2-TAMs contributed to immune evasion, metastasis, and treatment resistance in melanoma. He *et al.* [39] found that M2-TAMs promote resistance to both immunotherapy and chemotherapy in Gastric Cancer (GC) by inducing metabolic reprogramming and immune suppression. Specifically, gastric cancer cells upregulate interleukin-3 (IL-3) expression *via* the YAP1 signaling pathway, thereby driving macrophage M2 polarization. M2-TAMs secrete CCL8, which activates the JAK1/STAT3 signaling pathway in tumor cells, leading to GLUT3-dependent glycolytic reprogramming and enhancing resistance to 5-fluorouracil (5-FU). Chen *et al.* [40] reported that TAM-derived extracellular vesicles transfer GATA binding protein 3 (GATA3) into ovarian cancer cells, thereby activating the CD24/sialic acid-binding immunoglobulin-like lectin 10 (Siglec-10) axis and mediating immune evasion and chemoresistance. In summary, TAMs exhibit extensive heterogeneity in terms of origin, polarization, function, and distribution across different tumor types, and are closely associated with drug resistance (Fig. **2**).

Collectively, evidence from diverse tumor contexts reveals a convergent mechanistic framework in which M2-polarized TAMs function as central amplifiers of treatment resistance by orchestrating multiple interconnected pathways. At the cytokine level, IL-10 and TGF-β1 directly suppress effector T-cell activation and promote immune tolerance. At the chemokine level, the CCL8–JAK1/STAT3 axis drives metabolic reprogramming toward aerobic glycolysis, rendering tumor cells metabolically flexible and thus resistant to drugs such as 5-fluorouracil. At the post-transcriptional and epigenetic levels, METTL3-mediated m6A modification enhances the stability of immunosuppressive transcripts, while extracellular vesicle-mediated transfer of GATA3 upregulates checkpoint molecules such as CD24 and Siglec-10, further reinforcing immune evasion. Importantly, these pathways do not operate in isolation but rather converge to sustain a therapy-refractory state characterized by enhanced cancer stemness, impaired immune surveillance, and metabolic adaptability. This integrated understanding positions TAMs not merely as passive bystanders but as active orchestrators of the immunosuppressive niche, and underscores their potential as high-priority therapeutic targets in combination regimens designed to overcome multi-modal resistance mechanisms.

### 5.3 Strategies for Overcoming Tumor Immune Resistance Induced by TAMs

Since TAMs are a key factor in the treatment resistance of cancer, various targeted strategies have been developed to enhance anti-tumor immunity. These targeted strategies can help overcome tumor drug resistance. Li *et al.* [41] reported that controlling TAM infiltration can promote T cell infiltration, enhancing local and systemic delivery of anti-PD-1 antibody-conjugated platelets to inhibit postoperative recurrence. TAMs regulate T cell recruitment and abundance, contributing to anti-PD-1/PD-L1 resistance. Hence, targeting the immunosuppressive features of TAMs and increasing the abundance of anti-tumor CD^8+^T cells are feasible strategies to overcome treatment resistance in cancer [42]. Mechanistically, anti-angiogenic treatment is related to a high abundance of PD-1+T regulatory cells and TAMs in the TME. Depletion of TAMs using CSF1R inhibitor redirected PD-1 antibodies to CD^8+^T cells to improve the efficacy of combination therapy [43]. A recent study employed an alginate-based hydrogel nanoparticle loaded with pexidartinib (PLX) to release PLX at the tumor site and interfere with TAM infiltration. Previously, clodronate liposomes have been widely employed to deplete TAMs, and some anti-cancer chemotherapeutic agents, such as adriamycin and epirubicin, can also be employed to deplete TAMs [44]. In addition, the CCL2/CCR2 and CXCR4/CXCL12 signaling pathways play a key role in monocyte recruitment as well as TAM generation. Therefore, blocking the CCL2/CCR2 signaling pathway can restrict monocyte recruitment into tumor tissues, and inhibiting TAM infiltration by a CSF1 inhibitor can suppress tumor proliferation and migration. CXCR4 inhibitors, such as AMD3100, can be used to block the CXCR4/CXCL12 signaling axis [45]. Rodriguez-Garcia *et al.* [46] used chimeric antigen receptor T cells (CAR-T cells), selective clearance of folate receptor β (FRβ)-positive TAMs, and successfully controlled tumor progression in three different preclinical tumor models. Monoclonal antibodies alone are not effective in non-Hodgkin's lymphoma and often need to be combined with chemotherapy. In particular, the combination of the anti-CD47 monoclonal antibody Hu5F9-G4 with anti-CD20 monoclonal antibodies, such as rituximab, was more effective and safe in phase Ib clinical studies [47].

Macrophage polarization toward the M2 phenotype can prevent the infiltration of CD8+T cells into tumor tissues, inducing resistance to anti-PD-1/PD-L1 inhibitors. In a recent research, resistance to anti-PD-1 therapy was related to a high abundance of M2 macrophages in the clinical tissue samples of patients with advanced gastric cancer [48]. CSF-1/CSF-1R blockade reprogrammed TAMs toward the M1 phenotype, reduced granulin levels, and restored the anti-tumor response of CTLs. Accordingly, restoration of the anti-tumor immune response in granulin-depleted tumors enhanced treatment response to anti-PD-1 [49].

## 6 TREGS AND TUMOR IMMUNE RESISTANCE

### 6.1 The Mechanism by which Tregs Mediate Tumor Immune Resistance

Tregs, as an immunosuppressive subgroup of CD4+T cells, have a crucial role in maintaining tissue homeostasis as well as peripheral tolerance [50]. In TME, Tregs inhibit anti-tumor immune response and contribute to tumor immune evasion, which is closely related to resistance to immunotherapy as well as chemotherapy. In patients with NSCLC, low expression of phosphatase as well as tensin homolog deleted on chromosome ten (PTEN) was found to be associated with poor response to immunotherapy. PTEN-deficient tumor cells secrete high levels of TGF-β/CXCL10, which promotes the conversion of CD4+ lymphocytes into Tregs [51], thereby weakening anti-tumor immunity. Further functional studies [52] indicated that Tregs expressing CCR4 had stronger immunosuppressive function. These Tregs secrete high levels of IL-10 and IL-35, as well as other cytokines, which can inhibit the function of killer T cells and promote tumor immune escape. Zhang *et al.* [53] proposed that YTHDF2 is essential for Tregs in the TME and regulates the transcription of M6A-modified NF-кB negative regulators; thus, it can serve as a potential target for cancer immunotherapy. Shiri *et al.* [54] found that Treg-derived IL-10 upregulates PD-L1 expression in monocytes, which, in turn, reduces CD8+T cell infiltration and weakens the antitumor immune response in liver metastases of Colorectal Cancer (CRC). Future surveillance and management of liver metastases originating from colorectal cancer will be based on these findings [54]. Wen *et al.* [55] discovered that by controlling the Hsp70/stub1/Foxp3 pathway in Tregs, Salidroside (SAL) can suppress Treg function and lung cancer cell growth.

The reviewed evidence demonstrates that Tregs sustain immune resistance through a multi-tiered suppressive network that operates at both cellular and molecular levels. First, chemokine-driven recruitment, particularly *via* CCR4 and its cognate ligands, ensures sustained infiltration and accumulation of Tregs within the TME. Second, intracellular regulatory circuits, including YTHDF2-mediated m6A modification of NF-κB pathway regulators, stabilize and enhance the suppressive phenotype of intratumoral Tregs, maintaining their immunosuppressive capacity even under pro-inflammatory conditions. Third, Tregs exert paracrine immunosuppression by inducing PD-L1 expression on surrounding myeloid cells, thereby creating a bystander suppression effect that extends beyond direct Treg–T-cell contact. Notably, the functional output of these mechanisms is not additive but synergistic: Treg-derived IL-10 not only directly inhibits CD^8+^ T-cell infiltration and effector function but also reprograms monocytes to express higher levels of PD-L1, which in turn further suppresses residual T-cell activity. This cross-talk between Tregs and myeloid cells underscores the importance of viewing immune resistance as an integrated network phenomenon rather than a collection of isolated cellular defects. Therapeutic strategies that disrupt Treg recruitment, reprogram their function, or neutralize their suppressive mediators may therefore yield compounding benefits by simultaneously relieving multiple layers of immunosuppression and restoring effective anti-tumor immunity.

### 6.2 Strategies for Overcoming Tumor Immune Resistance by Targeting Tregs

The first strategy is to reduce Treg infiltration into tumor tissues. In a mouse model of colon cancer, treatment with tumor neoantigen vaccine and anti-PD-1, combined with the Treg modulator Bempegaldesleukin (BEMPEG) or anti-CTLA-4, led to tumor regression in almost all tumor-bearing animals [56]. Another study [57] used *L. gallinarum* combined with anti-PD-1 immunotherapy for colorectal cancer. It has been observed that indole-3-carboxylic acid (ICA) produced by Lactobacillus can inhibit Treg infiltration into the tumor and enhance the function of CD^8+^T cells, thereby improving the efficacy of immunotherapy. In addition, targeted inhibition of CCR4 was shown to significantly inhibit the recruitment of Tregs into tumor tissues and inhibit tumor growth. The combination of sorafenib and PD-1 inhibitors improved tumor drug resistance and enhanced the efficacy of immunotherapy [57, 58]. FEM@PFC is a novel immunomodulatory nanoagent whose perfluorocarbon (PFC) component can carry oxygen to the TME, alleviating tumor hypoxia and preventing Treg infiltration. More importantly, FEM@PFC depletion of glutathione effectively suppressed the immunosuppressive function of Tregs [59]. In pancreatic ductal adenocarcinoma, the combination of STAT3 Antisense Oligonucleotide (ASON) and radiotherapy promoted the immune response in the TME and throughout the body [60]. In addition, by targeting the Forkhead Box Protein P3 (FOXP3) gene, ASON effectively ameliorated the immunosuppressive function of Tregs, and its combination with immune checkpoint inhibitors enhanced the anti-tumor efficacy [61]. The second strategy is to induce Treg differentiation. Tregs selectively express glucocorticoid-induced tumor necrosis factor receptor (GITR) in glioblastoma. Targeting GITR with its activating antibody (αGITR) promoted the differentiation of CD4+ Tregs into Th1 cells, thereby alleviating Treg-mediated immunosuppression. Reprogrammed Tregs expressed the Th1 characteristic genes, produced interferon-gamma (IFN-γ), and exhibited cytotoxicity against tumor cells [62].

## 7 MYELOID-DERIVED SUPPRESSOR CELLS AND TUMOR IMMUNE RESISTANCE

### 7.1 Differentiation and Function of Myeloid-derived Suppressor Cells

MDSCs, as one of the most important types of stromal cells in the TME, are a multifunctional cell derived from bone marrow. MDSCs possess immunosuppressive properties and protect tumor cells against the host's immune system. Specifically, they produce ROS to inhibit T cell function [63, 64]. In normal circumstances, MDSCs differentiate into mature granulocytes, monocytes, or DCs in peripheral blood and tissues and participate in the immune response. However, their differentiation is inhibited in cancer or other pathological states, increasing the number of MDSCs and leading to persistent immunosuppression.

### 7.2 The Mechanism by which MDSCs Mediate Tumor Immune Resistance

MDSCs are involved in tumor immune resistance through multiple mechanisms. Firstly, they can directly or indirectly affect gene expression as well as signaling pathways in tumor cells by secreting cytokines, nitric oxide, ROS, and granulocyte-macrophage colony-stimulating factor (GM-CSF). Secondly, MDSCs can inhibit the proliferation as well as function of tumor-specific immune cells [65]. MDSCs can also induce T cell differentiation into Tregs and enhance immunosuppressive effects by secreting transforming growth factor-β (TGF-β) and IL-10 [66]. In addition, MDSCs reduce tumor sensitivity to chemotherapy and radiotherapy by promoting tumor stem cell differentiation. MDSCs can affect the overall environmental characteristics of TME. For example, MDSCs can enhance the low oxygen and low pH state of TME by secreting ROS, thus affecting drug stability and activity.

Synthesized evidence positions MDSCs as central orchestrators operating at the nexus of metabolic dysregulation, chronic inflammation, and stromal remodeling within the TME. Unlike other immunosuppressive populations that primarily act through direct cell-cell contact, MDSCs exert a dual effect: they directly inhibit T-cell and NK-cell function *via* secretion of ROS, nitric oxide, and arginase, while simultaneously reshaping the physicochemical properties of the TME itself. Specifically, MDSC-derived ROS and metabolic byproducts such as methylglyoxal exacerbate local hypoxia and acidification, creating a hostile microenvironment that compromises drug penetration, chemical stability, and biological activity. Furthermore, MDSCs promote the expansion of Tregs through secretion of TGF-β and IL-10, establishing a feed-forward loop of immunosuppression in which Tregs reciprocally support MDSC survival and function. This multi-dimensional activity—spanning metabolic reprogramming, immune cell paralysis, and environmental conditioning—explains why MDSC abundance often correlates with resistance to both chemotherapy and immunotherapy across diverse tumor types. Importantly, the heterogeneity of MDSC subsets (polymorphonuclear *vs*. monocytic MDSCs) adds another layer of complexity, as different subsets may dominate in distinct tumor contexts or at different stages of disease progression. Therapeutic strategies targeting MDSCs must therefore account for this functional and phenotypic diversity, and may benefit from combinatorial approaches that simultaneously induce MDSC differentiation, block recruitment, and neutralize suppressive mediators to achieve durable clinical benefit.

### 7.3 Strategies to Overcome MDSC-mediated Tumor Immune Resistance

Mei *et al.* [67] found that IL‑37 can inhibit tumorigenesis by inhibiting the immunosuppressive effects of MDSCs in the TME through metabolic reprogramming, highlighting its role as a novel avenue for cancer immunotherapy. Zhang *et al.* [68] found that C-C motif chemokine ligand 20 (CCL20) can regulate the TME to inhibit tumorigenesis, representing a novel strategy for cancer treatment. The first strategy is to promote the differentiation and maturation of MDSCs [69]. Promoting the differentiation of MDSCs into mature myeloid cells can weaken the immunosuppressive function of MDSCs. Vitamin D, all-trans retinoic acid, and vitamin A can regulate the transcription factors and related signaling pathways of MDSCs to induce their differentiation [70]. All-trans retinoic acid inhibits the generation and recruitment of MDSCs, thereby decreasing the number and activity of MDSCs in tumor tissues [71]. Gemcitabine, paclitaxel, and carboplatin can inhibit bone marrow proliferation and induce the apoptosis of MDSCs. Small-molecule inhibitors, such as CXCR2 inhibitors, CCR2 inhibitors, and CCR5 inhibitors, can also block the chemokine receptors of MDSCs and inhibit the migration of MDSCs [72]. In addition, the metabolism, energy supply, function, and signaling of MDSCs can be inhibited [73] to restore the activity of immune cells. Similarly, the glucose metabolism of MDSCs can also be inhibited by blocking glycolysis or the pentose phosphate pathways. Thus, analogs of specific glycolytic metabolites can reduce the abundance of MDSCs and reverse the immunosuppressive environment [74].

Therapeutic strategies targeting MDSCs have shown efficacy in a few animal models and clinical trials, but there are some challenges, such as the heterogeneity and plasticity of MDSCs, the identification and targeting of MDSCs, and the safety of therapeutic agents. Hence, it is necessary to understand the connection of MDSCs with tumor immune resistance and develop more effective and safer treatment strategies.

## 8 NATURAL KILLER CELLS AND TUMOR IMMUNE RESISTANCE

### 8.1 The Mechanisms by which Natural Killer Cells Mediate Tumor Immune Resistance

There is a complex interaction between Natural Killer (NK) cells and tumor drug resistance. An in-depth analysis of how NK cells induce tumor drug resistance and active exploration of new tumor treatment strategies based on NK cells will open new horizons for overcoming tumor drug resistance. Previous studies have found a group of tumor-associated NK cells with weakened anti-tumor function. A high abundance of these tumor-associated NK cells was shown to be associated with poor prognosis and resistance to immunotherapy [75]. In addition, other cell populations in the TME can inhibit the anti-tumor function of NK cells. The hypoxic environment induced by TME impairs multiple functions of NK cells, forming a favorable environment for tumor proliferation and metastasis. More importantly, tumor cells have stronger metabolic adaptability and nutrient uptake capacity than NK cells, which leads to resistance to CAR-NK therapy [76].

### 8.2 Strategies to Overcome Immune Resistance Mediated by NK Cells

Generally, NK cells can effectively inhibit the growth and spread of tumor cells and overcome the immune resistance of tumor cells [77]. There are three main strategies for enhancing NK cell killing function: the use of regulatory cytokines, adoptive NK cell therapy, and the use of engineered NK cells [78]. In addition, expanded NK cell-derived exosomes (eNK-EXO) from expanded NK cells can reverse the immunosuppressive state of NK cells, thereby enhancing anti-tumor activity [79]. Editing the destruction of TGF-β receptor II in NK cells and CAR-NK cells can help control refractory tumors. Tumor antigens targeted by CAR molecules are also expressed in NK cells. These antigens can also be specifically removed by gene editing to eliminate the interaction between NK cells and CAR-NK cells and enhance their killing effects on tumor cells [80]. The newly developed lipid nanoparticles loaded with stimulator of interferon genes (STING-LNP) not only induce anti-tumor immunity by activating NK cells but also exhibit synergistic effects with anti-PD-1 therapy [81].

## 9 OTHER IMMUNE CELLS, EXTRACELLULAR MATRIX, AND TUMOR IMMUNE RESISTANCE

In addition to TAMs, Tregs, MDSCs, and NK cells, ECM and Tumor-Associated Neutrophils (TANs) play a significant role in tumor progression as well as drug resistance.

### 9.1 Extracellular Matrix and Tumor Immune Resistance

The ECM supports the proliferation and metastasis of tumor cells. One significant non-cellular element of the TME that is essential to preserving tissue structure and function is the Extracellular Matrix (ECM). Drug distribution can be delayed or dissolved by the Extracellular Matrix (ECM), which serves as a physical barrier for the tumor [82]. ECM changes can influence the penetration as well as distribution of chemotherapeutic medications, reduce their effectiveness, and promote tumor cell invasion and metastasis.

TAMs have been linked to ECM composition and cancer treatment, according to Puttock *et al.* [83]. In ovarian cancer, they discovered that tumor ECM can rewire TAMs, which was linked to a poor prognosis. They also demonstrated how immune infiltration and immunotherapy sensitivity might be enhanced by targeting the extracellular matrix. *In vitro* and *in vivo*, type III Col causes tumor cell dormancy, according to Martino *et al.* [84] They showed that the shift from dormancy to reactivation was characterized by modifications in ECM Col orientation. As a chemotherapy sensitizer, Wang *et al.* [85] created an ECM deprivation system based on FN-targeted self-assembly peptide, which can effectively counteract the effects of ECM on tumor growth. This strategy may be a novel method to inhibit ECM as well as enhance the sensitivity to chemotherapeutic drugs.

### 9.2 Tumor-associated Neutrophils and Tumor Immune Resistance

By controlling TAN infiltration, Chan *et al.* [86] showed both *in vitro* and *in vivo* that pancreatic melatonin improved antitumor immunity in neuroendocrine tumors and pancreatic cancer. In their single-cell transcriptome investigation of 81 NSCLC patients, Hu *et al.* [87] discovered an overexpressed TAN-promoting cluster containing high mobility group box 1 (HMGB1), which was thought to inhibit tumor immunity and facilitate immune escape through the GATA2/HMGB1/TIM-3 axis. TANs infiltrating the tumor site can be divided into two subtypes: anti-tumor N1 TANs and pro-tumor N2 TANs. These TANs mainly mediate tumor immune resistance through neutrophil extracellular traps (NETs) [88]. NETs are widely distributed in various cancers and interact with stromal components, immune cells, and cancer cells. They reshape the stromal and extracellular environments in favor of cancer development, progression, and metastasis. Studies have shown that Net-associated Matrix Metalloproteinase9 (MMP9) is responsible for activating TGF-β secretion by tumor cells, which leads to epithelial-mesenchymal transformation and chemoresistance of tumor cells [89]. In addition, NETs affect the proliferation as well as activation of T cells and NK cells, limiting the efficacy of immunotherapy. There are two strategies to target TANs and overcome tumor immune resistance: 1) blocking TAN infiltration into the tumor tissue and preventing the local accumulation of TANs; 2) clearing TANs or modulating their differentiation into the N1 phenotype with anti-tumor function, while inhibiting their differentiation into the N2 phenotype with immunosuppressive function.

Evidence from studies on ECM remodeling and TAN-mediated neutrophil extracellular trap (NET) formation complements findings on TAMs, Tregs, MDSCs, and NK-cell dysfunction, collectively supporting an integrated model in which both cellular and non-cellular components of the TME co-evolve to sustain immune escape and treatment resistance. Specifically, ECM stiffness and composition not only serve as physical barriers to immune cell infiltration and drug penetration but also actively reprogram infiltrating macrophages toward immunosuppressive phenotypes, creating a bidirectional relationship between structural and cellular elements. Similarly, TANs do not function in isolation but rather interact with tumor cells, immune cells, and stromal components through NET formation, which activates TGF-β signaling, induces epithelial-mesenchymal transition, and suppresses T-cell and NK-cell proliferation. These structural and cellular mechanisms converge to establish a self-reinforcing ecosystem in which physical barriers limit immune access, immunosuppressive cells dampen effector function, and chronic inflammation further remodels the ECM to sustain this vicious cycle. Therapeutic strategies must therefore adopt a multi-modal approach that simultaneously targets structural elements (*e.g.*, through ECM-degrading enzymes or anti-fibrotic agents), neutralizes immunosuppressive cellular populations, and restores effector immune function. Only by addressing the full complexity of the TME—spanning molecular defects, cellular immunosuppression, and structural barriers—can we hope to achieve durable responses and overcome the pervasive challenge of treatment resistance in cancer.

### CONCLUSION AND FUTURE PROSPECTS

The mechanisms of immune resistance in the TME operate through a hierarchical cascade, with tumor-intrinsic molecular defects (such as TAP downregulation, MHC-I loss, and β2m mutations) serving as foundational drivers that precede and enable subsequent cell-mediated immune suppression. This mechanistic understanding highlights the critical importance of targeting both molecular defects and immunosuppressive cells for effective cancer immunotherapy. The balance between molecular-level and cell-level mechanisms in this review reflects the multifaceted nature of immune resistance and provides a comprehensive framework for understanding and overcoming therapeutic challenges.

Recent advances have elucidated dysfunctional immune components within the TME with increasing clarity. Many of these components are now targets for developing therapeutic drugs. Recently, with the advances in studies on TME, the use of immune cells has been proposed as a novel strategy to overcome tumor immune resistance. Clarifying the specific mechanism of immune cell-mediated tumor resistance in TME, as well as developing new strategies targeting corresponding cells, can advance cancer treatment. It is also worth mentioning that CAR-T cell technology and other adoptive cell therapies, as well as microenvironmental regulation targeting the TME matrix and TME metabolism, are important tools to overcome tumor immune resistance. Although the field faces many challenges, including the plasticity and heterogeneity of TAMs and MDSCs in the TME, we believe that targeting the TME is a promising approach to overcome tumor immune resistance.

## Figures and Tables

**Fig. (1):**
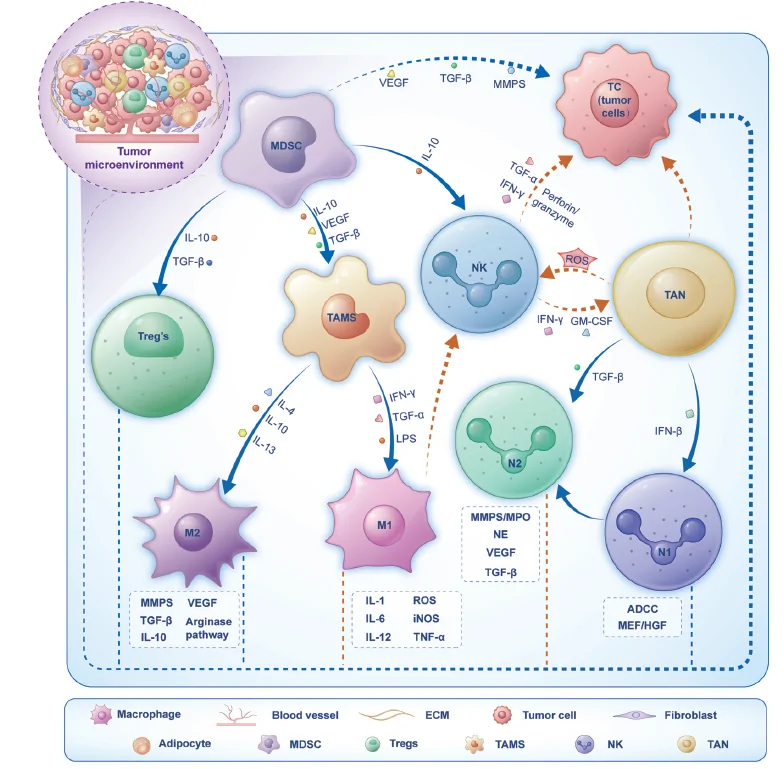
Components of the TME and immune cell interactions in antitumor responses. The yellow arrows represent antitumor effects, while the blue arrows indicate protumor effects.

**Fig. (2):**
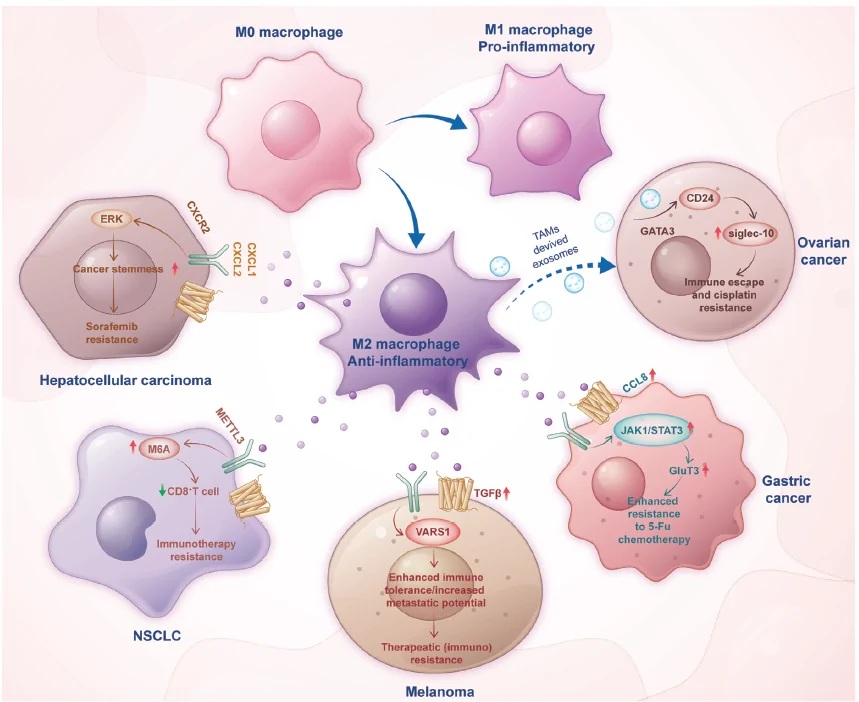
Heterogeneity of TAMs and mechanisms underlying TAM-mediated tumor drug resistance.

## LIST OF ABBREVIATIONS

ECM: Extracellular Matrix
MDR: Multi-drug Resistance
MDSCs: Myeloid-derived Suppressor Cells
TAMs: Tumor-associated Macrophages
TME: Tumor Microenvironment
Tregs: Regulatory T Cells

## References

[1] Jiang Y., Zhang H., Wang J., Liu Y., Luo T., Hua H. (2022). Targeting extracellular matrix stiffness and mechanotransducers to improve cancer therapy.. J. Hematol. Oncol..

[2] Hanahan D., Coussens L.M. (2012). Accessories to the crime: Functions of cells recruited to the tumor microenvironment.. Cancer Cell..

[3] La Rocca A., De Gregorio V., Lagreca E., Vecchione R., Netti P.A., Imparato G. (2023). Colorectal cancer bioengineered microtissues as a model to replicate tumor-ECM crosstalk and assess drug delivery systems in vitro.. Int. J. Mol. Sci..

[4] Anderson N.M., Simon M.C. (2020). The tumor microenvironment.. Curr. Biol..

[5] Castells M., Thibault B., Delord J.P., Couderc B. (2012). Implication of tumor microenvironment in chemoresistance: Tumor-associated stromal cells protect tumor cells from cell death.. Int. J. Mol. Sci..

[6] Dauer P., Nomura A., Saluja A., Banerjee S. (2017). Microenvironment in determining chemo-resistance in pancreatic cancer: Neighborhood matters.. Pancreatology.

[7] Liu Y., Liang J., Zhang Y., Guo Q. (2024). Drug resistance and tumor immune microenvironment: An overview of current understandings.. Int. J. Oncol..

[8] Sharma P., Hu-Lieskovan S., Wargo J.A., Ribas A. (2017). Primary, adaptive, and acquired resistance to cancer immunotherapy.. Cell..

[9] Majzner R.G., Mackall C.L. (2018). Tumor antigen escape from CAR T-cell therapy.. Cancer Discov..

[10] Singh V., Dwivedi S., Agrawal R., Ps M.R., Bansal A., Agarwal A., Misra S. (2025). Transporter associated with antigen processing proteins (TAP-1 and TAP-2) gene expression of MHC-I downregulated in oral squamous carcinoma.. Endocr. Metab. Immune Disord. Drug Targets.

[11] Hagel K.R., Arafeh R., Gang S., Arnoff T.E., Larson R.C., Doench J.G., Mathewson N.D., Wucherpfennig K.W., Maus M.V., Hahn W.C. (2023). Systematic interrogation of tumor cell resistance to chimeric antigen receptor T-cell therapy in pancreatic cancer.. Cancer Res..

[12] Restelli C., Ruella M., Paruzzo L., Tarella C., Pelicci P.G., Colombo E. (2024). Recent advances in immune-based therapies for acute myeloid leukemia.. Blood Cancer Discov..

[13] Nolan-Stevaux O., Li C., Liang L., Zhan J., Estrada J., Osgood T., Li F., Zhang H., Case R., Murawsky C.M., Estes B., Moore G.L., Bernett M.J., Muchhal U., Desjarlais J.R., Staley B.K., Stevens J., Cooke K.S., Aeffner F., Thomas O., Stieglmaier J., Lee J.L., Coxon A., Bailis J.M. (2024). AMG 509 (Xaluritamig), an anti-STEAP1 XmAb 2+1 T-cell redirecting immune therapy with avidity-dependent activity against prostate cancer.. Cancer Discov..

[14] Spranger S., Gajewski T.F. (2016). Tumor-intrinsic oncogene pathways mediating immune avoidance.. OncoImmunology.

[15] Neefjes J., Jongsma M.L.M., Paul P., Bakke O. (2011). Towards a systems understanding of MHC class I and MHC class II antigen presentation.. Nat. Rev. Immunol..

[16] McGranahan N., Rosenthal R., Hiley C.T., Rowan A.J., Watkins T.B.K., Wilson G.A., Birkbak N.J., Veeriah S., Van Loo P., Herrero J., Swanton C., Swanton C., Jamal-Hanjani M., Veeriah S., Shafi S., Czyzewska-Khan J., Johnson D., Laycock J., Bosshard-Carter L., Rosenthal R., Gorman P., Hynds R.E., Wilson G., Birkbak N.J., Watkins T.B.K., McGranahan N., Horswell S., Mitter R., Escudero M., Stewart A., Van Loo P., Rowan A., Xu H., Turajlic S., Hiley C., Abbosh C., Goldman J., Stone R.K., Denner T., Matthews N., Elgar G., Ward S., Costa M., Begum S., Phillimore B., Chambers T., Nye E., Graca S., Al Bakir M., Joshi K., Furness A., Ben Aissa A., Wong Y.N.S., Georgiou A., Quezada S., Hartley J.A., Lowe H.L., Herrero J., Lawrence D., Hayward M., Panagiotopoulos N., Kolvekar S., Falzon M., Borg E., Marafioti T., Simeon C., Hector G., Smith A., Aranda M., Novelli M., Oukrif D., Janes S.M., Thakrar R., Forster M., Ahmad T., Lee S.M., Papadatos-Pastos D., Carnell D., Mendes R., George J., Navani N., Ahmed A., Taylor M., Choudhary J., Summers Y., Califano R., Taylor P., Shah R., Krysiak P., Rammohan K., Fontaine E., Booton R., Evison M., Crosbie P., Moss S., Idries F., Joseph L., Bishop P., Chaturved A., Quinn A.M., Doran H., Leek A., Harrison P., Moore K., Waddington R., Novasio J., Blackhall F., Rogan J., Smith E., Dive C., Tugwood J., Brady G., Rothwell D.G., Chemi F., Pierce J., Gulati S., Naidu B., Langman G., Trotter S., Bellamy M., Bancroft H., Kerr A., Kadiri S., Webb J., Middleton G., Djearaman M., Fennell D., Shaw J.A., Le Quesne J., Moore D., Nakas A., Rathinam S., Monteiro W., Marshall H., Nelson L., Bennett J., Riley J., Primrose L., Martinson L., Anand G., Khan S., Amadi A., Nicolson M., Kerr K., Palmer S., Remmen H., Miller J., Buchan K., Chetty M., Gomersall L., Lester J., Edwards A., Morgan F., Adams H., Davies H., Kornaszewska M., Attanoos R., Lock S., Verjee A., MacKenzie M., Wilcox M., Bell H., Hackshaw A., Ngai Y., Smith S., Gower N., Ottensmeier C., Chee S., Johnson B., Alzetani A., Shaw E., Lim E., De Sousa P., Barbosa M.T., Bowman A., Jordan S., Rice A., Raubenheimer H., Proli C., Cufari M.E., Ronquillo J.C., Kwayie A., Bhayani H., Hamilton M., Bakar Y., Mensah N., Ambrose L., Devaraj A., Buderi S., Finch J., Azcarate L., Chavan H., Green S., Mashinga H., Nicholson A.G., Lau K., Sheaff M., Schmid P., Conibear J., Ezhil V., Ismail B., Irvin-sellers M., Prakash V., Russell P., Light T., Horey T., Danson S., Bury J., Edwards J., Hill J., Matthews S., Kitsanta Y., Suvarna K., Fisher P., Keerio A.D., Shackcloth M., Gosney J., Postmus P., Feeney S., Asante-Siaw J., Aerts H.J.W.L., Dentro S., Dessimoz C. (2017). Allele-specific HLA loss and immune escape in lung cancer evolution.. Cell..

[17] Campoli M., Ferrone S. (2008). HLA antigen changes in malignant cells: Epigenetic mechanisms and biologic significance.. Oncogene.

[18] Spranger S., Bao R., Gajewski T.F. (2015). Melanoma-intrinsic β-catenin signalling prevents anti-tumour immunity.. Nature.

[19] Garrido F., Algarra I., García-Lora A.M. (2010). The escape of cancer from T lymphocytes: Immunoselection of MHC class I loss variants harboring structural-irreversible “hard” lesions.. Cancer Immunol. Immunother.

[20] Abele R., Tampé R. (2004). The ABCs of immunology: Structure and function of TAP, the transporter associated with antigen processing.. Physiology.

[21] Rock K.L., York I.A., Goldberg A.L. (2004). Post-proteasomal antigen processing for major histocompatibility complex class I presentation.. Nat. Immunol..

[22] Padariya M., Kote S., Mayordomo M., Dapic I., Alfaro J., Hupp T., Fahraeus R., Kalathiya U. (2022). Corrigendum to “Structural determinants of peptide-dependent TAP1-TAP2 transit passage targeted by viral proteins and altered by cancer-associated mutations”.. Comput. Struct. Biotechnol. J..

[23] Bui J.D., Schreiber R.D. (2007). Cancer immunosurveillance, immunoediting and inflammation: Independent or interdependent processes?.. Curr. Opin. Immunol..

[24] Wang H., Liu B., Wei J. (2021). Beta2-microglobulin(B2M) in cancer immunotherapies: Biological function, resistance and remedy.. Cancer Lett..

[25] Zaretsky J.M., Garcia-Diaz A., Shin D.S., Escuin-Ordinas H., Hugo W., Hu-Lieskovan S., Torrejon D.Y., Abril-Rodriguez G., Sandoval S., Barthly L., Saco J., Homet Moreno B., Mezzadra R., Chmielowski B., Ruchalski K., Shintaku I.P., Sanchez P.J., Puig-Saus C., Cherry G., Seja E., Kong X., Pang J., Berent-Maoz B., Comin-Anduix B., Graeber T.G., Tumeh P.C., Schumacher T.N.M., Lo R.S., Ribas A. (2016). Mutations associated with acquired resistance to PD-1 blockade in melanoma.. N. Engl. J. Med..

[26] Zhou M., Sacirbegovic F., Zhao K., Rosenberger S., Shlomchik W.D. (2020). T cell exhaustion and a failure in antigen presentation drive resistance to the graft-versus-leukemia effect.. Nat. Commun..

[27] Sade-Feldman M., Jiao Y.J., Chen J.H., Rooney M.S., Barzily-Rokni M., Eliane J.P., Bjorgaard S.L., Hammond M.R., Vitzthum H., Blackmon S.M., Frederick D.T., Hazar-Rethinam M., Nadres B.A., Van Seventer E.E., Shukla S.A., Yizhak K., Ray J.P., Rosebrock D., Livitz D., Adalsteinsson V., Getz G., Duncan L.M., Li B., Corcoran R.B., Lawrence D.P., Stemmer-Rachamimov A., Boland G.M., Landau D.A., Flaherty K.T., Sullivan R.J., Hacohen N. (2017). Resistance to checkpoint blockade therapy through inactivation of antigen presentation.. Nat. Commun..

[28] Lorenzi S., Forloni M., Cifaldi L., Antonucci C., Citti A., Boldrini R., Pezzullo M., Castellano A., Russo V., van der Bruggen P., Giacomini P., Locatelli F., Fruci D. (2012). IRF1 and NF-kB restore MHC class I-restricted tumor antigen processing and presentation to cytotoxic T cells in aggressive neuroblastoma.. PLoS One.

[29] Chim L.K., Williams I.L., Bashor C.J., Mikos A.G. (2023). Tumor-associated macrophages induce inflammation and drug resistance in a mechanically tunable engineered model of osteosarcoma.. Biomaterials.

[30] Liu Y., Li C., Lu Y., Liu C., Yang W. (2022). Tumor microenvironment-mediated immune tolerance in development and treatment of gastric cancer.. Front. Immunol..

[31] Labani-Motlagh A., Ashja-Mahdavi M., Loskog A. (2020). The tumor microenvironment: A Milieu Hindering and obstructing antitumor immune responses.. Front. Immunol..

[32] Wang H., Tian T., Zhang J. (2021). Tumor-associated macrophages (TAMs) in colorectal cancer (CRC): From mechanism to therapy and prognosis.. Int. J. Mol. Sci..

[33] Xiao M., He J., Yin L., Chen X., Zu X., Shen Y. (2021). Tumor-associated macrophages: Critical players in drug resistance of breast cancer.. Front. Immunol..

[34] Zaghdoudi S., Decaup E., Belhabib I., Samain R., Cassant-Sourdy S., Rochotte J., Brunel A., Schlaepfer D., Cros J., Neuzillet C., Strehaiano M., Alard A., Tomasini R., Rajeeve V., Perraud A., Mathonnet M., Pearce O.M.T., Martineau Y., Pyronnet S., Bousquet C., Jean C. (2020). FAK activity in cancer‐associated fibroblasts is a prognostic marker and a druggable key metastatic player in pancreatic cancer.. EMBO Mol. Med..

[35] Khalaf K., Hana D., Chou J.T.T., Singh C., Mackiewicz A., Kaczmarek M. (2021). Aspects of the tumor microenvironment involved in immune resistance and drug resistance.. Front. Immunol..

[36] Wang H.C., Haung L.Y., Wang C.J., Chao Y.J., Hou Y.C., Yen C.J., Shan Y.S. (2022). Tumor-associated macrophages promote resistance of hepatocellular carcinoma cells against sorafenib by activating CXCR2 signaling.. J. Biomed. Sci..

[37] Wu L., Cheng D., Yang X., Zhao W., Fang C., Chen R., Ji M. (2022). M2-TAMs promote immunoresistance in lung adenocarcinoma by enhancing METTL3-mediated m6A methylation.. Ann. Transl. Med..

[38] Wu Z., Lei K., Li H., He J., Shi E. (2022). Transcriptome-based network analysis related to M2-like tumor-associated macrophage infiltration identified VARS1 as a potential target for improving melanoma immunotherapy efficacy.. J. Transl. Med..

[39] He Z., Chen D., Wu J., Sui C., Deng X., Zhang P., Chen Z., Liu D., Yu J., Shi J., Li G., Yao X. (2021). Yes associated protein 1 promotes resistance to 5-fluorouracil in gastric cancer by regulating GLUT3-dependent glycometabolism reprogramming of tumor-associated macrophages.. Arch. Biochem. Biophys..

[40] Chen C., Zhang L., Ruan Z. (2023). GATA3 encapsulated by tumor-associated macrophage-derived extracellular vesicles promotes immune escape and chemotherapy resistance of ovarian cancer cells by upregulating the CD24/Siglec-10 axis.. Mol. Pharm..

[41] Li Z., Ding Y., Liu J., Wang J., Mo F., Wang Y., Chen-Mayfield T.J., Sondel P.M., Hong S., Hu Q. (2022). Depletion of tumor associated macrophages enhances local and systemic platelet-mediated anti-PD-1 delivery for post-surgery tumor recurrence treatment.. Nat. Commun..

[42] House I.G., Savas P., Lai J., Chen A.X.Y., Oliver A.J., Teo Z.L., Todd K.L., Henderson M.A., Giuffrida L., Petley E.V., Sek K., Mardiana S., Gide T.N., Quek C., Scolyer R.A., Long G.V., Wilmott J.S., Loi S., Darcy P.K., Beavis P.A. (2020). Macrophage-derived CXCL9 and CXCL10 are required for antitumor immune responses following immune checkpoint blockade.. Clin. Cancer Res..

[43] Martinez-Usatorre A., Kadioglu E., Boivin G., Cianciaruso C., Guichard A., Torchia B., Zangger N., Nassiri S., Keklikoglou I., Schmittnaegel M., Ries C.H., Meylan E., De Palma M. (2021). Overcoming microenvironmental resistance to PD-1 blockade in genetically engineered lung cancer models.. Sci. Transl. Med..

[44] Xia Y., Rao L., Yao H., Wang Z., Ning P., Chen X. (2020). Engineering macrophages for cancer immunotherapy and drug delivery.. Adv. Mater..

[45] Zhang H., Liu L., Liu J., Dang P., Hu S., Yuan W., Sun Z., Liu Y., Wang C. (2023). Roles of tumor-associated macrophages in anti-PD-1/PD-L1 immunotherapy for solid cancers.. Mol. Cancer.

[46] Rodriguez-Garcia A., Lynn R.C., Poussin M., Eiva M.A., Shaw L.C., O’Connor R.S., Minutolo N.G., Casado-Medrano V., Lopez G., Matsuyama T., Powell D.J. (2021). CAR-T cell-mediated depletion of immunosuppressive tumor-associated macrophages promotes endogenous antitumor immunity and augments adoptive immunotherapy.. Nat. Commun..

[47] Advani R., Flinn I., Popplewell L., Forero A., Bartlett N.L., Ghosh N., Kline J., Roschewski M., LaCasce A., Collins G.P., Tran T., Lynn J., Chen J.Y., Volkmer J.P., Agoram B., Huang J., Majeti R., Weissman I.L., Takimoto C.H., Chao M.P., Smith S.M. (2018). CD47 blockade by Hu5F9-G4 and Rituximab in Non-Hodgkin’s Lymphoma.. N. Engl. J. Med..

[48] Wu C., Qiu Y., Zhang R., Li X., Liang H., Wang M., Li F., Zhu M., Ye G., Liu H., Li G., Zhao L. (2022). Association of peripheral basophils with tumor M2 macrophage infiltration and outcomes of the anti-PD-1 inhibitor plus chemotherapy combination in advanced gastric cancer.. J. Transl. Med..

[49] Quaranta V., Rainer C., Nielsen S.R., Raymant M.L., Ahmed M.S., Engle D.D., Taylor A., Murray T., Campbell F., Palmer D.H., Tuveson D.A., Mielgo A., Schmid M.C. (2018). Macrophage-derived granulin drives resistance to immune checkpoint inhibition in metastatic pancreatic cancer.. Cancer Res..

[50] Sakaguchi S., Sakaguchi N., Asano M., Itoh M., Toda M. (1995). Immunologic self-tolerance maintained by activated T cells expressing IL-2 receptor α-chains (CD25). Breakdown of a single mechanism of self-tolerance causes various autoimmune diseases.. J. Immunol..

[51] Exposito F., Redrado M., Houry M., Hastings K., Molero-Abraham M., Lozano T., Solorzano J.L., Sanz-Ortega J., Adradas V., Amat R., Redin E., Leon S., Legarra N., Garcia J., Serrano D., Valencia K., Robles-Oteiza C., Foggetti G., Otegui N., Felip E., Lasarte J.J., Paz-Ares L., Zugazagoitia J., Politi K., Montuenga L., Calvo A. (2023). PTEN loss confers resistance to anti–PD-1 therapy in non–small cell lung cancer by increasing tumor infiltration of regulatory T cells.. Cancer Res..

[52] Gao Y., You M., Fu J., Tian M., Zhong X., Du C., Hong Z., Zhu Z., Liu J., Markowitz G.J., Wang F.S., Yang P. (2022). Intratumoral stem-like CCR4+ regulatory T cells orchestrate the immunosuppressive microenvironment in HCC associated with hepatitis B.. J. Hepatol..

[53] Zhang L., Dou X., Zheng Z., Ye C., Lu T.X., Liang H.L., Wang L., Weichselbaum R.R., He C. (2023). YTHDF2/m6 A/NF-κB axis controls anti-tumor immunity by regulating intratumoral Tregs.. EMBO J..

[54] Shiri A.M., Zhang T., Bedke T., Zazara D.E., Zhao L., Lücke J., Sabihi M., Fazio A., Zhang S., Tauriello D.V.F., Batlle E., Steglich B., Kempski J., Agalioti T., Nawrocki M., Xu Y., Riecken K., Liebold I., Brockmann L., Konczalla L., Bosurgi L., Mercanoglu B., Seeger P., Küsters N., Lykoudis P.M., Heumann A., Arck P.C., Fehse B., Busch P., Grotelüschen R., Mann O., Izbicki J.R., Hackert T., Flavell R.A., Gagliani N., Giannou A.D., Huber S. (2024). IL-10 dampens antitumor immunity and promotes liver metastasis via PD-L1 induction.. J. Hepatol..

[55] Wen Z., Liu T., Zhang Y., Yue Q., Meng H., He Y., Yang Y., Li M., Zheng J., Lin W. (2023). Salidroside regulates tumor microenvironment of non-small cell lung cancer via Hsp70/Stub1/Foxp3 pathway in Tregs.. BMC Cancer.

[56] D'Alise A.M., Leoni G., De Lucia M., Langone F., Nocchi L., Tucci F.G., Micarelli E., Cotugno G., Troise F., Garzia I., Vitale R., Bignone V., Di Matteo E., Bartolomeo R., Charych D.H., Lahm A., Zalevsky J., Nicosia A., Scarselli E. (2021). Maximizing cancer therapy via complementary mechanisms of immune activation: PD-1 blockade, neoantigen vaccination, and Tregs depletion.. J. Immunother Cancer.

[57] Fong W., Li Q., Ji F., Liang W., Lau H.C.H., Kang X., Liu W., To K.K.W., Zuo Z., Li X., Zhang X., Sung J.J.Y., Yu J. (2023). Lactobacillus gallinarum-derived metabolites boost anti-PD1 efficacy in colorectal cancer by inhibiting regulatory T cells through modulating IDO1/Kyn/AHR axis.. Gut.

[58] Imbert C., Montfort A., Fraisse M., Marcheteau E., Gilhodes J., Martin E., Bertrand F., Marcellin M., Burlet-Schiltz O., Peredo A.G., Garcia V., Carpentier S., Tartare-Deckert S., Brousset P., Rochaix P., Puisset F., Filleron T., Meyer N., Lamant L., Levade T., Ségui B., Andrieu-Abadie N., Colacios C. (2020). Resistance of melanoma to immune checkpoint inhibitors is overcome by targeting the sphingosine kinase-1.. Nat. Commun..

[59] Li Z., Deng Y., Sun H., Tan C., Li H., Mo F., Wang Y., Li J., Zhou Z., Sun M. (2023). Redox modulation with a perfluorocarbon nanoparticle to reverse Treg-mediated immunosuppression and enhance anti-tumor immunity.. J. Control. Release.

[60] Piper M., Van Court B., Mueller A., Watanabe S., Bickett T., Bhatia S., Darragh L.B., Mayeda M., Nguyen D., Gadwa J., Knitz M., Corbo S., Morgan R., Lee J.J., Dent A., Goodman K., Messersmith W., Schulick R., Del Chiaro M., Zhu Y., Kedl R.M., Lenz L., Karam S.D. (2022). Targeting treg-expressed STAT3 enhances NK-mediated surveillance of metastasis and improves therapeutic response in pancreatic adenocarcinoma.. Clin. Cancer Res..

[61] Revenko A., Carnevalli L.S., Sinclair C., Johnson B., Peter A., Taylor M., Hettrick L., Chapman M., Klein S., Solanki A., Gattis D., Watt A., Hughes A.M., Magiera L., Kar G., Ireland L., Mele D.A., Sah V., Singh M., Walton J., Mairesse M., King M., Edbrooke M., Lyne P., Barry S.T., Fawell S., Goldberg F.W., MacLeod A.R. (2022). Direct targeting of FOXP3 in tregs with AZD8701, a novel antisense oligonucleotide to relieve immunosuppression in cancer.. J. Immunother Cancer.

[62] Amoozgar Z., Kloepper J., Ren J., Tay R.E., Kazer S.W., Kiner E., Krishnan S., Posada J.M., Ghosh M., Mamessier E., Wong C., Ferraro G.B., Batista A., Wang N., Badeaux M., Roberge S., Xu L., Huang P., Shalek A.K., Fukumura D., Kim H.J., Jain R.K. (2021). Targeting Treg cells with GITR activation alleviates resistance to immunotherapy in murine glioblastomas.. Nat. Commun..

[63] Lv B., Wang Y., Ma D., Cheng W., Liu J., Yong T., Chen H., Wang C. (2022). Immunotherapy: Reshape the tumor immune microenvironment.. Front. Immunol..

[64] Zhang W., Li S., Li C., Li T., Huang Y. (2022). Remodeling tumor microenvironment with natural products to overcome drug resistance.. Front. Immunol..

[65] Cimen Bozkus C., Elzey B.D., Crist S.A., Ellies L.G., Ratliff T.L. (2015). Expression of cationic amino acid transporter 2 is required for myeloid-derived suppressor cell–mediated control of T cell immunity.. J. Immunol..

[66] Baumann T., Dunkel A., Schmid C., Schmitt S., Hiltensperger M., Lohr K., Laketa V., Donakonda S., Ahting U., Lorenz-Depiereux B., Heil J.E., Schredelseker J., Simeoni L., Fecher C., Körber N., Bauer T., Hüser N., Hartmann D., Laschinger M., Eyerich K., Eyerich S., Anton M., Streeter M., Wang T., Schraven B., Spiegel D., Assaad F., Misgeld T., Zischka H., Murray P.J., Heine A., Heikenwälder M., Korn T., Dawid C., Hofmann T., Knolle P.A., Höchst B. (2020). Regulatory myeloid cells paralyze T cells through cell–cell transfer of the metabolite methylglyoxal.. Nat. Immunol..

[67] Mei Y., Zhu Y., Yong K.S.M., Hanafi Z.B., Gong H., Liu Y., Teo H.Y., Hussain M., Song Y., Chen Q., Liu H. (2024). IL-37 dampens immunosuppressive functions of MDSCs via metabolic reprogramming in the tumor microenvironment.. Cell. Rep..

[68] Zhang R., Dong M., Tu J., Li F., Deng Q., Xu J., He X., Ding J., Xia J., Sheng D., Chang Z., Ma W., Dong H., Zhang Y., Zhang L., Zhang L., Liu S. (2023). PMN-MDSCs modulated by CCL20 from cancer cells promoted breast cancer cell stemness through CXCL2-CXCR2 pathway.. Signal. Transduct. Target Ther..

[69] Nefedova Y., Nagaraj S., Rosenbauer A., Muro-Cacho C., Sebti S.M., Gabrilovich D.I. (2005). Regulation of dendritic cell differentiation and antitumor immune response in cancer by pharmacologic-selective inhibition of the janus-activated kinase 2/signal transducers and activators of transcription 3 pathway.. Cancer Res..

[70] Ni X., Hu G., Cai X. (2019). The success and the challenge of all-trans retinoic acid in the treatment of cancer.. Crit. Rev. Food. Sci. Nutr..

[71] Dolcetti L., Peranzoni E., Ugel S., Marigo I., Fernandez Gomez A., Mesa C., Geilich M., Winkels G., Traggiai E., Casati A., Grassi F., Bronte V. (2010). Hierarchy of immunosuppressive strength among myeloid‐derived suppressor cell subsets is determined by GM‐CSF.. Eur. J. Immunol..

[72] Millrud C.R., Bergenfelz C., Leandersson K. (2017). On the origin of myeloid-derived suppressor cells.. Oncotarget.

[73] Fujita M., Kohanbash G., Fellows-Mayle W., Hamilton R.L., Komohara Y., Decker S.A., Ohlfest J.R., Okada H. (2011). COX-2 blockade suppresses gliomagenesis by inhibiting myeloid-derived suppressor cells.. Cancer Res..

[74] Jian S.L., Chen W.W., Su Y.C., Su Y.W., Chuang T.H., Hsu S.C., Huang L.R. (2017). Glycolysis regulates the expansion of myeloid-derived suppressor cells in tumor-bearing hosts through prevention of ROS-mediated apoptosis.. Cell. Death Dis..

[75] Tang F., Li J., Qi L., Liu D., Bo Y., Qin S., Miao Y., Yu K., Hou W., Li J., Peng J., Tian Z., Zhu L., Peng H., Wang D., Zhang Z. (2023). A pan-cancer single-cell panorama of human natural killer cells.. Cell..

[76] Li L., Mohanty V., Dou J., Huang Y., Banerjee P.P., Miao Q., Lohr J.G., Vijaykumar T., Frede J., Knoechel B., Muniz-Feliciano L., Laskowski T.J., Liang S., Moyes J.S., Nandivada V., Basar R., Kaplan M., Daher M., Liu E., Li Y., Acharya S., Lin P., Shanley M., Rafei H., Marin D., Mielke S., Champlin R.E., Shpall E.J., Chen K., Rezvani K. (2023). Loss of metabolic fitness drives tumor resistance after CAR-NK cell therapy and can be overcome by cytokine engineering.. Sci. Adv..

[77] Myers J.A., Miller J.S. (2021). Exploring the NK cell platform for cancer immunotherapy.. Nat. Rev. Clin. Oncol..

[78] Fantini M., Arlen P.M., Tsang K.Y. (2023). Potentiation of natural killer cells to overcome cancer resistance to NK cell-based therapy and to enhance antibody-based immunotherapy.. Front. Immunol..

[79] Luo H., Zhou Y., Zhang J., Zhang Y., Long S., Lin X., Yang A., Duan J., Yang N., Yang Z., Che Q., Yang Y., Guo T., Zi D., Ouyang W., Yang W., Zeng Z., Zhao X. (2023). NK cell-derived exosomes enhance the anti-tumor effects against ovarian cancer by delivering cisplatin and reactivating NK cell functions.. Front. Immunol..

[80] Valeri A., García-Ortiz A., Castellano E., Córdoba L., Maroto-Martín E., Encinas J., Leivas A., Río P., Martínez-López J. (2022). Overcoming tumor resistance mechanisms in CAR-NK cell therapy.. Front. Immunol..

[81] Nakamura T., Sato T., Endo R., Sasaki S., Takahashi N., Sato Y., Hyodo M., Hayakawa Y., Harashima H. (2021). STING agonist loaded lipid nanoparticles overcome anti-PD-1 resistance in melanoma lung metastasis via NK cell activation.. J. Immunother Cancer.

[82] Bigos K.J.A., Quiles C.G., Lunj S., Smith D.J., Krause M., Troost E.G.C., West C.M., Hoskin P., Choudhury A. (2024). Tumour response to hypoxia: Understanding the hypoxic tumour microenvironment to improve treatment outcome in solid tumours.. Front. Oncol..

[83] Puttock E.H., Tyler E.J., Manni M., Maniati E., Butterworth C., Burger Ramos M., Peerani E., Hirani P., Gauthier V., Liu Y., Maniscalco G., Rajeeve V., Cutillas P., Trevisan C., Pozzobon M., Lockley M., Rastrick J., Läubli H., White A., Pearce O.M.T. (2023). Extracellular matrix educates an immunoregulatory tumor macrophage phenotype found in ovarian cancer metastasis.. Nat. Commun..

[84] Di Martino J.S., Nobre A.R., Mondal C., Taha I., Farias E.F., Fertig E.J., Naba A., Aguirre-Ghiso J.A., Bravo-Cordero J.J. (2021). A tumor-derived type III collagen-rich ECM niche regulates tumor cell dormancy.. Nat. Can.

[85] Wang L., Li C., Wang J., Yang G., Lv Y., Fu B., Jian L., Ma J., Yu J., Yang Z., Wu P., Li G., Liu X., Kang Z., Wang Z., Wang L., Wang H., Xu W. (2022). Transformable ECM deprivation system effectively suppresses renal cell carcinoma by reversing anoikis resistance and increasing chemotherapy sensitivity.. Adv. Mater..

[86] Chan Y., Tan H., Lu Y., Zhang C., Cheng C., Wu J., Wang N., Feng Y. (2023). Pancreatic melatonin enhances anti-tumor immunity in pancreatic adenocarcinoma through regulating tumor-associated neutrophils infiltration and NETosis.. Acta. Pharm. Sin B.

[87] Hu Q., Wang R., Zhang J., Xue Q., Ding B. (2023). Tumor-associated neutrophils upregulate PANoptosis to foster an immunosuppressive microenvironment of non-small cell lung cancer.. Cancer Immunol. Immunother.

[88] Liu Z., Dou Y., Lu C., Han R., He Y. (2025). Neutrophil extracellular traps in tumor metabolism and microenvironment.. Biomark. Res..

[89] Mousset A., Lecorgne E., Bourget I., Lopez P., Jenovai K., Cherfils-Vicini J., Dominici C., Rios G., Girard-Riboulleau C., Liu B., Spector D.L., Ehmsen S., Renault S., Hego C., Mechta-Grigoriou F., Bidard F.C., Terp M.G., Egeblad M., Gaggioli C., Albrengues J. (2023). Neutrophil extracellular traps formed during chemotherapy confer treatment resistance via TGF-β activation.. Cancer Cell..

